# Loss of connexin 36 elicits abnormalities in thalamocortical network activity relevant to neuropsychiatric disorders

**DOI:** 10.1038/s41398-026-04018-1

**Published:** 2026-04-25

**Authors:** James M. McNally, Sean Carey, David S. Uygun, Stephen Thankachan, Radhika Basheer

**Affiliations:** 1 VA Boston Healthcare System and Harvard Medical School, Dept. of Psychiatry, West Roxbury, MA USA; 2 School of Life Sciences, University of Warwick, Coventry, UK

**Keywords:** Physiology, Neuroscience, Schizophrenia

## Abstract

Neuronal gap junctions, or electrical synapses, are extensively expressed in the mammalian forebrain and play a key role in synchronizing network activity. Connexin 36 (**Cx36**) is the primary gap junction protein in mature GABAergic neurons, but its contribution to thalamocortical oscillations and cognitive processes remains unclear. Here, we examined the effects of Cx36 deletion on sleep/wake regulation, spontaneous and evoked EEG activity, and behavior in mice. While Cx36 knockout (**KO**) mice displayed largely intact sleep architecture, spectral analysis revealed impaired gamma and beta band activity and reduced sigma power surges preceding NREM–REM transitions. Spindle density was preserved, but spindle amplitude and duration were reduced. Cx36KO mice exhibited blunted gamma responses to ketamine, impaired 40 Hz auditory steady-state responses, and reduced mismatch negativity with attenuated ERP amplitudes and altered evoked power. Behaviorally, Cx36KO mice showed impaired social habituation and reduced investigation-induced gamma activity. These findings demonstrate that Cx36-containing gap junctions are essential for maintaining thalamocortical synchrony and support translational EEG biomarkers relevant to schizophrenia and other psychiatric disorders. Cx36 may therefore represent a novel therapeutic target for modulating dysfunctional network activity in neuropsychiatric disease.

## Introduction

Rhythmogenesis and oscillatory activity of neuronal networks are vital to the central nervous system function [1]. Aberrant oscillatory activity corresponds to dysfunctional network connectivity and may underlie pathological symptoms associated with a wide range of neurological and neuropsychiatric disorders such as Schizophrenia, Alzheimer’s Disease, autism spectrum disorder, and Parkinson’s Disease [2–6]. Thus, it is critical to develop a better understanding of the neural circuitry behind the generation of such oscillatory activity to help derive novel targets for therapeutic intervention.

Gap junctions are specialized intercellular channels that allow direct electrical and metabolic communication between adjacent cells [7]. Often overshadowed by chemical synapses, gap junctions constitute a fundamental mechanism of neural communication and are present in most circuits responsible for shaping behavior [8–11]. Electrical synaptic connectivity between neurons is believed to be critical for synchronization of neuronal activity in the brain and plays a significant role in the modulation of neuronal oscillatory activity and network function [8, 12–16].

Connexin36 (Cx36) is the predominant gap junction protein which forms electrical synapses in the mammalian central nervous system [15, 17]. Extensive investigations have used Cx36 constitutive gene knock out mice which exhibit almost complete (~ 95%) loss of neuronal gap junction coupling [11, 18, 19]. These mice demonstrate impaired high frequency network activity, including gamma band activity [12, 20], deficient neuronal synchronization, including reduced synchronization between cortical interneurons, high-frequency ripples and β-oscillations in the hippocampus [20], and loss of synchronized activity in the inferior olivary nucleus and the thalamic reticular nucleus [11, 18, 19].

While the role of Cx36 in cortical and hippocampal oscillatory activity has been explored, its effects on behavioral state regulation and evoked/task-associated cortical oscillatory activity have not yet been investigated. Electrical synapses are thought to be modifiable, frequency and activity-dependent determinants of brain function, particularly in state regulation of the thalamocortical system [21, 22]. Changes in frequency of large-scale oscillations are a determinant of changes in states of behavior and awareness [22]. Consequently, electrical synapses could serve as a modifiable resonance feedback system that fine tunes such oscillations in neuronal networks, including the thalamocortical system [23, 24]. Here we utilize Cx36KO mice to examine the effects of loss of Cx36 containing gap junctions on sleep/wake behavior, state specific spontaneous cortical oscillatory activity, and stimulus evoked oscillatory activity.

## Methods and materials

### Ethical approval and animal handling

For this study, Adult (> 4 months) male and female Cx36 KO mice and wild-type littermate controls (FBV background) were housed in standard SPF facilities at 21 °C with 12:12 h light/dark cycle (lights-on 7:00AM), and food and water available ad libitum. Genotyped mice were a gracious gift from Barry W. Connors’ group at Brown University. All procedures were performed in strict accordance with National Institutes of Health guidelines and were approved by the VA Boston Institutional Animal Care and Use Committee (Project #1736856). This committee and the associated animal facility are fully accredited by the Association for Assessment and Accreditation of Laboratory Animal Care (AAALAC). In this work, all efforts were made to minimize the number of experimental animals used and number of procedures to limit animal suffering and distress.

### Stereotaxic surgeries

Under isoflurane anesthesia (induction, 5%; maintenance, 1–2%), EEG screw electrodes (0.10”, Cat No. 8403, Pinnacle Technology Inc.) were implanted bilaterally above frontal cortex (AP 1.5 mm; ML ± 1 mm). Reference and ground screws were placed in the bone above the midline cerebellum and parietal cortex, respectively. Electromyography (EMG) electrodes were placed in the nuchal muscle. All electrodes were connected to a head mount (Cat No. 8402, Pinnacle Technology Inc.) which was affixed to the skull with dental cement. Each mouse was allowed to recover from surgery for at least one week prior to experimental use.

### In vivo electrophysiology

EEG/EMG signals were amplified and recorded using a 3 Channel-EEG System (#8200-K1-SL, Pinnacle Technologies) at a 2000 Hz sampling rate. For auditory stimulation experiments, WinWCP (John Dempster, University of Strathclyde) or Spike2 (CED) software along with a secondary digitizer (Digidata 1440, Molecular Devices or 1401, CED) were used to both record EEG/EMG and generate output signals to control stimuli. For the ASSR task, trains of white noise clicks were generated by delivering digital output pulses (5 V square wave, 10 ms pulse width) to a cage-mounted speaker. For the mismatched negativity (MMN) task, pure tone signals were generated by delivering sinusoidal analog output signals, generated in MATLAB, to cage mounted speaker. An audio amplifier and dB meter were used to ensure that auditory stimuli were delivered at an appropriate decibel level (80 dB).

### Sleep staging and NREM-REM sigma surge analysis

Sleep-wake staging was scored manually using commercial software (SleepSign, Kissei Comtec) in 5 s epochs using the following criteria: wakefulness was defined by desynchronized low voltage fast EEG with high muscle movements in EMG; NREM sleep was characterized by high-amplitude low-frequency slow wave/delta band EEG activity and decreased muscle tone, and REM sleep was defined by enhanced theta band (4–7 Hz) and low voltage fast activity coupled with a complete loss of muscle tone and rapid eye movements [25]. Custom MATLAB scripts were then utilized for state-based power spectral analysis of EEG activity and bout analysis of sleep/wake behavior.

Time-frequency spectrograms for state-transitions were generated and analyzed in a manner similar to that described in Uygun et al. [26]. We first down-sampled EEG records to 40 Hz and screened for outliers, replacing values > 10 standard deviations away from the mean with zeros. We then identified timepoints corresponding to NREM to REM transitions, defined as adjacent epochs where the animals scored state changed from NREM to REM, and then extracted EEG data which was continuously scored as NREM and REM surrounding this timepoint (+/- 70 s). Multi-taper time frequency analysis was then performed (Chronux Toolbox; Chronux.org) using 5 tapers with a 10 s sliding window in 100 ms steps. Time frequency spectrograms were then computed for each transition for each mouse and averaged. Each within-animal averaged time-frequency spectrum was then normalized to its average power from wakefulness (I.E. Frequency bin from spectra/Frequency bin from wake). The resulting spectra were grand averaged by mean across all the animals in the group. Averaged power in the sigma band (10–15 Hz) was plotted as lines with standard error envelopes, smoothed by a 5 s moving average.

### Social habituation task

For this task, experimental mice were first tethered and then habituated to an open field arena (44 × 44 cm) containing an empty circular stimulus chamber (10 cm diameter) in one corner for a period of 10 min. Mice were then removed from the chamber and placed back in their home cage for 5 min. During this time, a gender matched novel mouse was placed inside the stimulus chamber in the open field arena. The experimental mice were then reintroduced to the arena and allowed to explore for 5 min. This process was repeated 4 times, with 5 min intertrial periods in the home cage.

The mouse remained tethered throughout this entire procedure, and EEG/EMG activity was recorded during task performance. An automated on-line video tracking system (EthoVision XT, Noldus Information Tech.) was used for real time monitoring of experimental mouse nose point, mid-point and tail during task performance. This system was used to timestamp EEG records at timepoints corresponding to the onset of the experimental mouse investigating the novel mouse (mouse nose < 2 cm from stimulus cage). All investigation timepoints derived from automated detection were manually confirmed post hoc. This software was also used for automated analysis of behavioral performance, including investigation frequency (number of times the mouse midpoint was detected to be < 5 cm from stimulus cage perimeter) and total distance traveled. This automated system increased throughput and allowed us to mitigate experimenter bias.

For social investigation associated EEG data, multi-taper power spectral analysis [27] was performed on 6 s segments of EEG data (2 s pre- & 4 s post-investigation onset). The power values in the time frequency spectra from each investigation were then normalized to the mean power in each frequency bin across the 2 s preceding the investigation. These normalized spectra of total power for each investigation were then averaged across all mice for each phase of the task. For statistical analysis of this data, power across the frequency band of interest (30–55 Hz) for each 6 s EEG segment was binned (500 ms segments) and values compared between groups using a repeated measure analysis of variance (RMANOVA). We chose to focus on this frequency range based on prior work which suggested that activity in this range reflects top-down predictive coding during a spatial working memory tests [28], and our own published findings [29] using similar behavioral tasks showing prominent increases in power in this range associated with object investigation.

### Data and statistical analyses

Data were analyzed offline using custom scripts written for MATLAB (R2022b, MathWorks). Analysis of spectral power and/or phase was performed using either the multi-taper method [27], or complex Morlet wavelet analysis, as described previously [29]. All data are presented as mean ± standard error. Sample size was chosen to be consistent with prior studies using similar experimental designs and outcome measures, which have reliably detected effects of comparable magnitude. This sample size has been shown empirically to provide adequate power for detecting biologically meaningful effects while maintaining statistical rigor. All collected data was included in analysis, except in circumstances where LFP signals were degraded. Given the population of experimental mice for this study, no randomization was employed regarding allocation of mice to experimental groups. For all experiments, variance between groups appeared comparable based on inspection of data distributions and summary statistics. Statistical analyses were performed using both JMPpro12 (SAS Institute Inc.) and R (R project.org). Comparisons of means between 2 groups were evaluated using 2-tailed Mann Whitney U tests. Multiple mean comparisons were performed using RMANOVA and Mann Whitney post-hoc analysis. The Kolmogorov–Smirnov test (K–S test) was used for comparison of probability distributions. Power spectra were evaluated using the Jackknifing U-statistical test to mitigate effects of artifacts and intermittent outliers in data [30]. All data and analysis scripts used in this manuscript are available upon reasonable request.

## Results

### Connexin 36 KO minimally affects sleep/wake but alters state specific high frequency oscillatory activity

State regulation and switching between sleep and wakefulness or between inattentive and vigilant states, requires shifts in network function and modulation of systems involved in sensory processing [31–35]. Given the hypothesized role of Cx36 containing electrical synapses in facilitating thalamocortical synchronized neural activity [22], we initially sought to determine the impact of the loss of Cx36 on sleep/wake behavior. To assess this, 24 h of EEG activity were analyzed from both Cx36KO mice and wild-type (WT) littermate controls (see Table 1). Overall, we observed that Cx36KO mice (n = 7) did not show any significant alteration from WT (n = 7) in the total amount of time spent in wake, NREM or REM across both the light and dark periods. Despite this, we found a significant decrease in the number of wakefulness bouts (WT 121.1 ± 2.9; KO 80.6 ± 7.0; 2- tailed Mann Whitney U Test: z = 2.68, p = 0.01) with a concomitant significant increase in bout duration (in seconds, WT: 121.9 ± 11.9; KO: 186.59 ± 21.9; 2- tailed Mann Whitney U Test: z = −2.69, p = 0.01) during the light period, but not the dark period. We additionally observed significant decreases in REM bout duration (WT: 90.0 ± 3.4, KO: 71.5 ± 5.4; 2- tailed Mann Whitney U Test: z = 2.38, p = 0.02) in Cx36KO mice during the dark period.

**Table 1:** Sleep staging data and bout analysis from 24 h of EEG data.

		Wake		NREM		REM	
		Lights On	Lights Off	Lights On	Lights Off	Lights On	Lights Off
**Total Time**	**WT**	39.6 ± 1.6%	55.6 ± 2.9%	52.5 ± 1.3%	39.6 ± 2.4%	7.9 ± 0.5%	4.7 ± 0.7%
	**KO**	39.1 ± 2.2%	52.9 ± 2.1%	52.8 ± 2.0%	43.2 ± 2.0%	8.1 ± 0.5%	3.9 ± 0.4%
**Bout #**	**WT**	**121.1** **±** **2.9**	92.1 ± 9.4	255.3 ± 32.3	164.0 ± 11.5	40.1 ± 4.7	21.0 ± 1.6
	**KO**	**80.6** **±** **7.0**	78.6 ± 5.9	232.6 ± 12.5	180.6 ± 18.2	45.6 ± 8.4	21.7 ± 3.9
**Bout Duration**	**WT**	**121.9** **±** **11.9**	253.2 ± 26.3	91.55 ± 12.1	100.1 ± 11.7	76.6 ± 2.3	**90.0** **±** **3.4**
	**KO**	**186.59** **±** **21.9**	276.9 ± 34.4	95.5 ± 6.2	102.0 ± 9.6	72.0 ± 4.7	**71.5** **±** **5.4**

Bold text represent significantly different values (2-way Mann Whitney U test; p < 0.05)

### Overall impact of Cx36 KO on power spectral density across states

Power spectral density was analyzed to determine if Cx36KO mice showed state specific alteration in specific oscillatory bands (see Table 2; WT: n = 6; Cx36KO: n = 7; one WT mouse was omitted from this analysis due to poor signal quality). As expected from prior rodent studies, we observed significant deficits in spontaneous high frequency oscillatory activity in the frontal cortex [20, 36, 37]. This included significant impairments in low frequency gamma band activity (30 - 55 Hz) during REM across both the light (2- tailed Mann Whitney U Test: z = 2.11, p = 0.03) and dark (2- tailed Mann Whitney U Test: z = 2.29, p = 0.02) periods. Low frequency gamma band activity was also significantly decreased for KO mice in wake during the dark period (2- tailed Mann Whitney U Test: z = 2.11, p = 0.03) and showed a trend level decrease during the light period (2- tailed Mann Whitney U Test: z = 1.79, p = 0.07). We additionally observed a significant reductions in high frequency gamma band activity (65 - 100 Hz) during REM in the dark period (2- tailed Mann Whitney U Test: z = 2.50, p = 0.01) and a trend-level decrease in beta band activity (15 – 25 Hz) during REM in the light period (2- tailed Mann Whitney U Test: z = 1.79, p = 0.07). Finally, we observed a trend-level increase in low frequency theta activity specific to the wake during the light period (2- tailed Mann Whitney U Test: z = −1.64, p = 0.10).

**Table 2:** Power spectral density analysis of Sleep/Wake EEG data.

		Wake		NREM		REM	
		Lights On	Lights Off	Lights On	Lights Off	Lights On	Lights Off
**SWA (0.5–2** **Hz)**	**WT**	0.101 ± 0.005	0.102 ± 0.007	0.139 ± 0.007	0.143 ± 0.005	0.081 ± 0.008	0.073 ± 0.010
	**KO**	0.106 ± 0.006	0.107 ± 0.006	0.139 ± 0.007	0.141 ± 0.005	0.081 ± 0.006	0.073 ± 0.004
**Delta (0.5–4** **Hz)**	**WT**	0.336 ± 0.013	0.332 ± 0.011	0.393 ± 0.011	0.431 ± 0.016	0.262 ± 0.015	0.250 ± 0.023
	**KO**	0.329 ± 0.013	0.326 ± 0.007	0.399 ± 0.006	0.438 ± 0.017	0.254 ± 0.016	0.246 ± 0.016
**Low Theta (5–7** **Hz)**	**WT**	*0.179* ± *0.005*	0.198 ± 0.003	0.162 ± 0.002	0.174 ± 0.002	0.231 ± 0.005	0.201 ± 0.012
	**KO**	*0.193* ± *0.004*	0.209 ± 0.016	0.171 ± 0.008	0.178 ± 0.009	0.241 ± 0.019	0.202 ± 0.013
**High Theta (7–9** **Hz)**	**WT**	0.114 ± 0.005	0.131 ± 0.005	0.089 ± 0.002	0.095 ± 0.002	0.162 ± 0.008	0.151 ± 0.004
	**KO**	0.116 ± 0.007	0.126 ± 0.004	0.093 ± 0.004	0.097 ± 0.003	0.154 ± 0.012	0.155 ± 0.017
**Sigma (10–15** **Hz)**	**WT**	0.090 ± 0.004	0.092 ± 0.004	0.107 ± 0.007	0.104 ± 0.005	0.127 ± 0.006	0.113 ± 0.009
	**KO**	0.092 ± 0.007	0.094 ± 0.005	0.096 ± 0.003	0.101 ± 0.005	0.113 ± 0.007	0.107 ± 0.006
**Beta (15–25** **Hz)**	**WT**	0.066 ± 0.003	0.067 ± 0.004	0.052 ± 0.003	0.057 ± 0.004	*0.080* ± *0.003*	0.074 ± 0.006
	**KO**	0.067 ± 0.007	0.069 ± 0.006	0.049 ± 0.002	0.055 ± 0.003	*0.066* ± *0.006*	0.066 ± 0.005
**Low Gamma (30–55** **Hz)**	**WT**	*0.064* *±* *0.005*	**0.069** ± **0.005**	0.028 ± 0.002	0.029 ± 0.002	**0.060** ± **0.006**	**0.059** ± **0.006**
	**KO**	*0.048* *±* *0.005*	**0.050** ± **0.005**	0.024 ± 0.003	0.024 ± 0.003	**0.039** ± **0.004**	**0.038** ± **0.002**
**High Gamma (65–100** **Hz)**	**WT**	0.014 ± 0.001	0.017 ± 0.001	0.004 ± 0.0003	0.005 ± 0.0004	0.011 ± 0.001	**0.011** ± **0.001**
	**KO**	0.012 ± 0.002	0.013 ± 0.002	0.004 ± 0.001	0.003 ± 0.001	0.007 ± 0.002	**0.006** ± **0.001**

Bold text represent significantly different values (2-way Mann Whitney U test; p < 0.05).

Italicized text represents trend-level difference in values (2-way Mann Whitney U test; p < 0.10).

### Loss of Cx36 impairs sleep spindles and NREM to REM transition sigma surge

Cx36 containing electrical synapses have been shown to be critical for coordination of synchronous activity in the thalamic reticular nucleus [23, 38]. Synchronized firing of inhibitory neurons in the thalamic reticular nucleus is believed to be primarily responsible for the generation of sleep spindles, which are transient oscillatory events which occur in the sigma frequency band specifically during NREM sleep. Prior studies have shown that blocking electrical coupling via halothane can abolish spindle activity [39]. In Cx36KO mice, we observed no significant changes in spontaneous sigma band power across all states and time periods investigated, compared to controls. This finding is similar to prior studies which showed that constitutive loss of channels which are crucial to the production of sleep spindles did not impact spontaneous sigma activity [40, 41]. However, this does not rule out other more subtle effects of Cx36 loss on sleep spindle activity. Therefore, we wanted to focus specifically on how loss of Cx36 impacts sleep spindles.

**Fig. 1:**
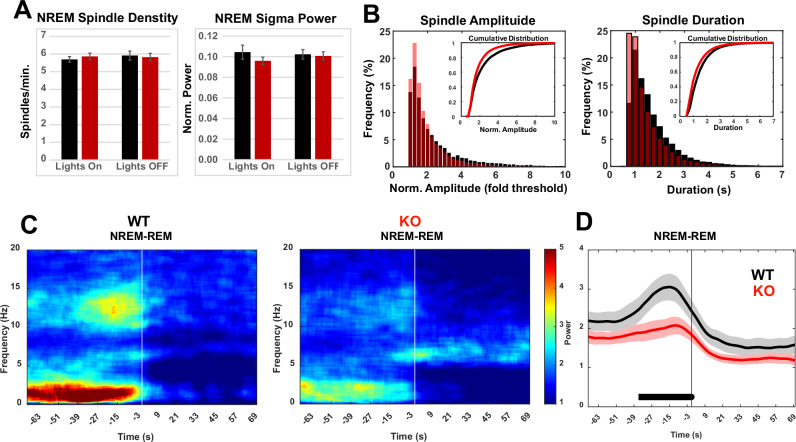
Sleep spindle and sigma abnormalities in Cx36KO mice. **A** Spindle density was unchanged between genotypes. **B** Amplitude and duration were significantly reduced in KO mice. **C** Sigma surge at NREM–REM transitions was present in WT but absent in KO mice. **D** Quantification confirmed reduced sigma power in KO (*p* < 0.05).

Here, we utilized an automated spindle detection algorithm to assess spindle activity in Cx36KO (n = 7) mice vs WT (n = 6) littermate controls [42]. This analysis revealed no significant change in NREM sleep spindle density across both the light and dark periods (Fig. 1A). Despite this, we observed significant reductions in both the normalized amplitude (Cx36KO: 2.01 ± 0.08; WT: 2.81 ± 0.16; K-S Test: 0.058, p < 0.001) and duration (Cx36KO: 1.47 ± 0.05; WT: 1.96 ± 0.07; K-S Test: 0.079, p < 0.001) of sleep spindles in Cx36KO mice (Fig. 1B).

Additionally, sleep spindles have been observed to be particularly pronounced just prior to transitions from NREM to REM state, resulting in a so called “surge” in sigma band power during this time [26, 40]. We examined power spectral activity specifically during these state transitions and observed a significant suppression of sigma surge during these spindle rich periods, in Cx36KO mice compared to WT controls (Fig. 1 C). To assess this reduction in sigma power at NREM-REM transition we summed area under the sigma power curve (Fig. 1D) between −48 s and 0 s (i.e., the transition to REM). Compared with WT sigma power (3.07 ± 0.25), Cx36KO sigma power (2.01 ± 0.21) was significantly reduced (2- tailed Mann Whitney U test, z = 2.11, p = 0.02), with a percentage change of −24.95 ± 14.04%.

### Loss of Cx36 impairs both pharmacologically induced and stimulus evoked gamma band activity

**Fig. 2:**
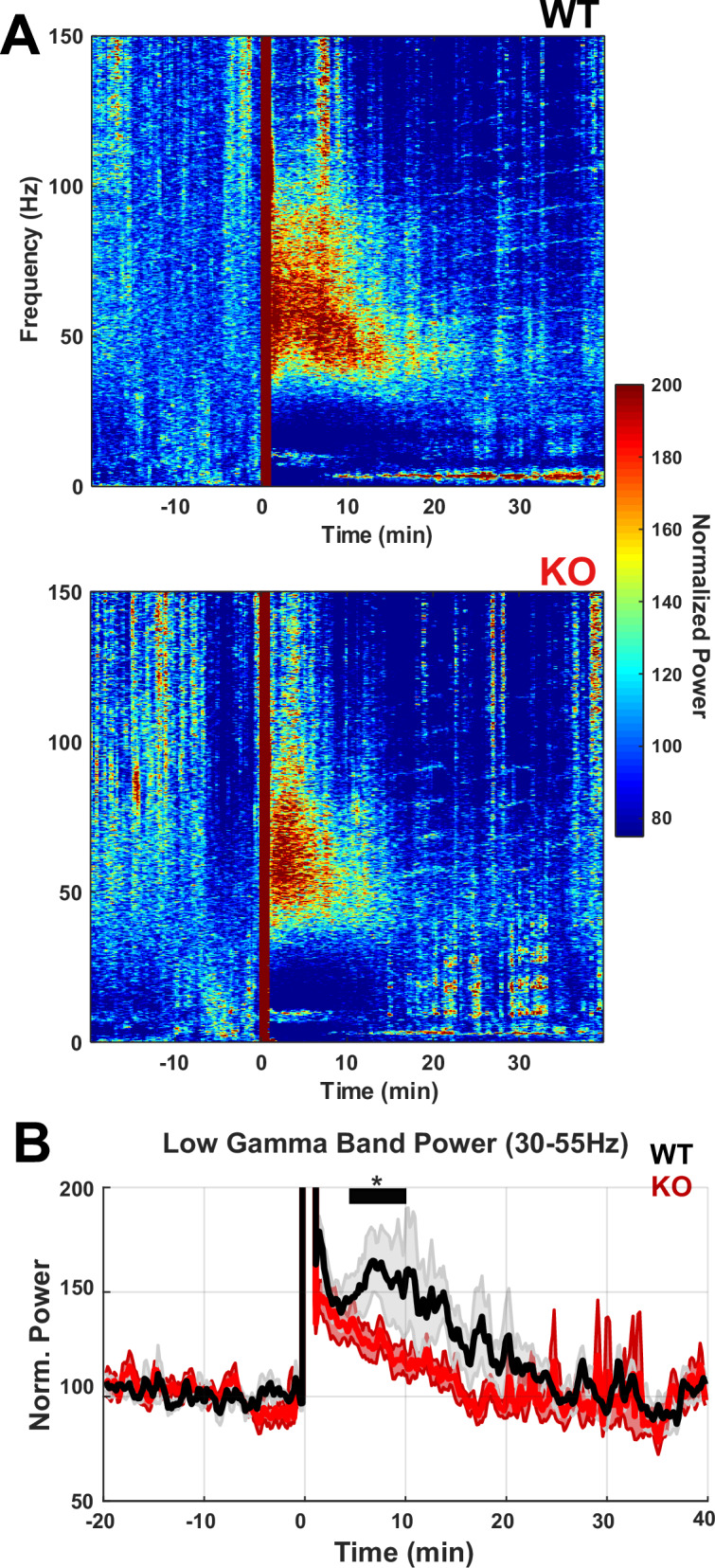
Ketamine-evoked gamma activity is blunted in Cx36KO mice. **A** Time–frequency plots show ketamine-induced broadband gamma increases in WT and KO. **B** KO mice exhibited significantly reduced low gamma power compared with WT (*p* < 0.05).

Consistent with prior published work, we observed that spontaneous gamma band activity is impaired in Cx36KO mice (see Table 2) [19, 20, 36]. To explore this finding further, we next examined how the loss of Cx36 expression impacts both pharmacologically induced cortical gamma band activity and auditory-evoked steady state gamma band activity in Cx36KO mice. We first investigated the effects of Cx36KO on increased broadband gamma power induced by the psychotomimetic drug ketamine. To test this, frontal cortex EEG activity was monitored for 60 min in both Cx36KO (n = 6) mice and WT controls (n = 6). Following a 20 min habituation period after tethering, mice received an acute subanesthetic dose of ketamine (15 mg/kg; I.P.). As predicted by previous work, acute injection of ketamine rapidly elevated broadband gamma band power in both WT and Cx36KO mice (Fig. 2A) [29, 43]. To compare the effects between these two groups, we measured both low (30–55 Hz) and high (65–80 Hz) gamma band activity between 5–10 min post injection (see Fig. 2B) this value was then normalized to power in the same frequency band at baseline (5 min period prior to injection). Overall, we observed a significant reduction in ketamine induced low frequency gamma band activity in Cx36KO mice compared to WT (Cx36KO: 120.02 ± 6.11; WT: 151.97 ± 14.97; 2- tailed Mann Whitney U test, z = 2.23, p = 0.03), while high frequency gamma band activity was not significantly changed (Cx36KO: 116.20. ± 9.55; WT: 150.56 ± 20.70; 2- tailed Mann Whitney U test, z = 0.85, p = 0.39).

**Fig. 3:**
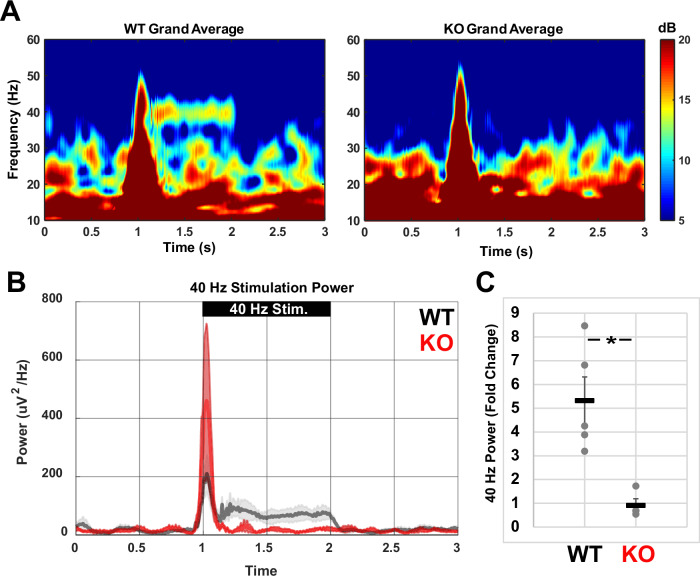
Impaired 40 Hz auditory steady-state response (ASSR) in Cx36KO mice. **A** Representative spectrograms ASSR to 40 Hz auditory click trains in WT and KO mice. **B–C** KO mice showed reduced 40 Hz evoked power relative to WT (*p* < 0.05).

Second, we utilized the auditory steady state response (ASSR) paradigm to assess the effects of the loss of Cx36 on stimulus evoked steady state oscillatory activity. Synchronization of cortical neuron firing in response to auditory stimuli represents a process related to cognitive function and is consistently impaired across a number of psychiatric disorders [44, 45]. Such synchronous activity is likely to be facilitated by electrical synaptic connectivity [46]. For ASSR, both Cx36KO (n = 4) and WT (n = 5) control mice were exposed to repeated bouts of auditory stimuli, consisting of 1 s trains of audible clicks ( ~ 80 dB) delivered at 40 Hz. ASSR was assessed from the averaged cortical EEG response to 100 repetitions of the auditory stimulus train (repeated every 2 s). Power spectral density analysis was then used to examine the evoked power at the stimulus frequency (40 ± 5 Hz). Comparing the pre-stimulus 40 Hz power to that observed during the auditory stimulation, we observed that the Cx36KO mice showed a significant impairment in 40 Hz ASSR (Fig. 3; Cx36KO: −8.59 ± 27.43 fold change from baseline; WT: 432.17 ± 99.72-fold change; 2- tailed Mann Whitney U test, z = 2.41, p = 0.02).

### Cx36KO exhibit impaired mismatch negativity response

**Fig. 4:**
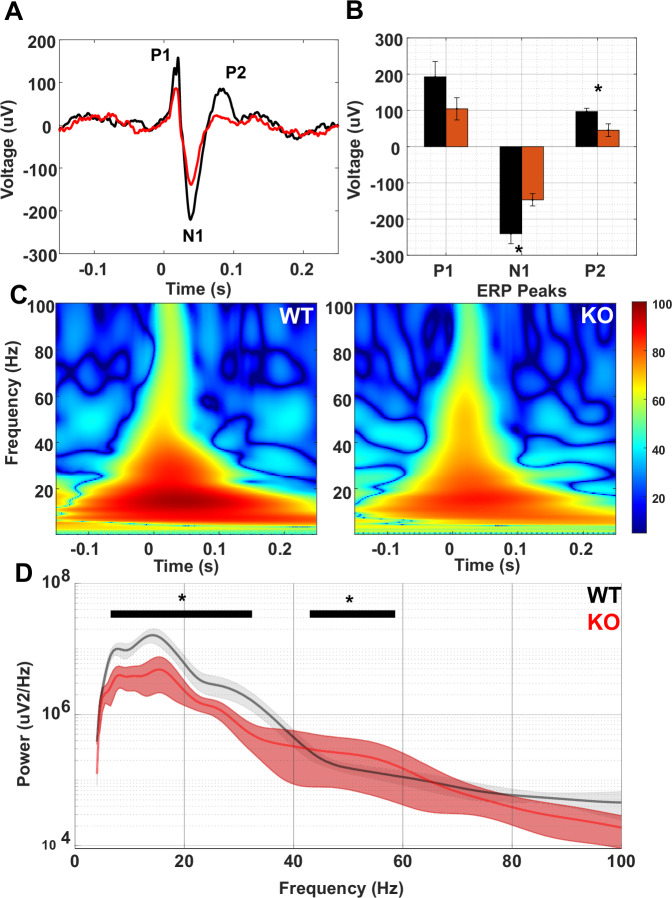
Altered mismatch negativity (MMN) in Cx36KO mice. **A** Average differential ERPs (deviant–standard tones) in WT and KO mice. **B** KO mice exhibited reduced N1 and P2 amplitudes. **C–D** Evoked power was significantly decreased at 6–35 Hz and increased at 43–61 Hz in KO mice (*p* < 0.05).

The mismatch negativity (MMN) task has similarly been described as a translationally relevant biomarker for understanding and treating psychotic disorders such as schizophrenia [47, 48]. This task examines the brain’s response to violations of a rule, established by a sequence of sensory stimuli which can result in a change in sensory-evoked event related potentials (ERP). To examine the impact of the loss of Cx36 on MMN in mice, experimental animals were exposed to a train of 100 ms auditory stimuli (80 dB) consisting of 90% 2.5 kHz tones (standard stimuli) and 10% 8 kHz tones (deviant stimuli), presented with an inter-stimulus interval of 1 s. The train consisted of 1000 total stimuli (~ 20 min total duration). Averaged EEG response to standard and deviant tones were then calculated providing averaged event related potentials (ERP) for both standard and deviant tones in each block. The standard ERP was then subtracted from the deviant to provide a differential ERP for both Cx36KO (n = 5) and WT (n = 5) mice (Fig. 4A). We observed 3 distinct peaks in the differential ERP; this included 2 positive peaks (P1 & P2) with a negative peak in between (N1) (Fig. 4A). The amplitude of both positive and negative ERP peaks were then calculated and compared between WT and Cx36KO mice to assess effects of the loss of Cx36 (Fig. 4B). While there was no significant change in the initial positive peak (P1; Cx36KO: 102.90 ± 37.21 µV ; WT: 200.25 ± 43.71 µV; 2- tailed Mann Whitney U test, z = 1.67, p = 0.09), Cx36KO mice showed a significant decrease in the amplitude of the negative peak (N1; Cx36KO: −133.87 ± 26.73 µV ; WT: −243.65 ± 28.45 µV; 2- tailed Mann Whitney U test, z = −2.15, p = 0.03) and trend level decrease in the second positive peak (P2; Cx36KO: 50.51 ± 17.34 µV ; WT: 108.75 ± 9.03 µV; 2- tailed Mann Whitney U test, z = 1.91, p = 0.05). Spectral analysis of resultant ERPs was then performed to assay evoked power across different frequency bands in WT versus Cx36KO mice (Fig. 4C). Here we observed a significant decrease in power between 6 and 35 Hz and a significant elevation in power between 43–61 Hz (Fig. 4D; Jackknifing U-Statistic: p < 0.05). Overall, our data shows that Cx36KO mice exhibit impaired MMN response with both impaired ERP peak amplitude as well as impaired evoked power.

### Cx36 loss impairs social cognition and social investigation-evoked gamma band activity

**Fig. 5:**
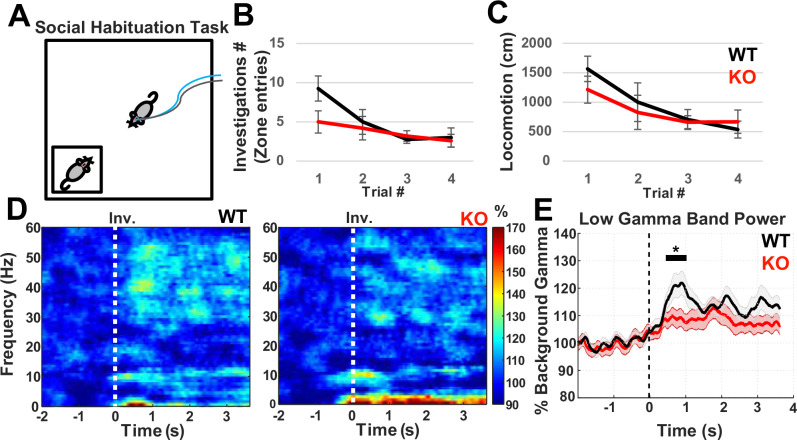
Impaired social habituation and task-evoked gamma in Cx36KO mice. **A** Experimental design of repeated social habituation task. **B** WT mice habituated across trials, whereas KO did not. **C** Locomotion was unchanged between groups. **D–E** KO mice showed reduced gamma power during social investigation (*p* < 0.05).

Deficits in social cognition are common across a number of psychiatric disorders [49–51]. To examine the impact of the loss of Cx36 on this process we employed the social habituation task, which assesses the natural propensity of rodents to habituate to an increasingly familiar cage-mate [52]. For this task, a novel mouse was placed in a stimulus cage within an open field area. Experimental mice were then placed in the arena for 5 min and allowed to investigate a novel mouse (Fig. 5A). This was repeated four consecutive times for each mouse. Video tracking was used to monitor animal behavior and analyze task performance. We initially examined the frequency of social investigations across each trial for wildtype (n = 4) versus Cx36KO (n = 5) mice. As shown in Fig. 5B, wildtype mice exhibit the predicted decrease in investigation frequency across the course of the four trials indicative of habituation to the novel mouse. However, Cx36KO mice did not exhibit this habituation. Our analysis showed a significant difference between groups (RMANOVA, Genotype*Trial, F3,21 = 4.26, p = 0.02) and a trend level decrease in investigations for trial #1 for KO mice compared to WT, (2-tailed Mann Whitney U test, z = 1.73, p = 0.08, Fig. 5B). This effect was not mediated by a lack of trial engagement as we did not observe any significant difference in locomotion between WT and KO mice (Fig. 5C).

We additionally recorded frontal EEG activity to directly examine the effects of the loss of Cx36 expression on task-evoked neural activity during this task. We performed spectral analysis on EEG activity recorded during social investigations in both WT and Cx36KO mice during performance of the task (Fig. 5D). We observed an apparent decrease in investigation elicited low gamma band power. For quantitative analysis of this data, low gamma power was summed across each investigation and then divided into 250 ms bins for each investigation. We then averaged the data across all investigations across the 4 trials (Fig. 5E) for both WT (n = 4 mice,105 investigations) and Cx36KO (n = 5 mice, 86 investigations) mice. Here we observed a significant decrease in power in the investigation evoked response in Cx36KO mice in bins representing between 0.5 s and 1 s following an observed social investigation (2-tailed Mann Whitney U test, z = 2.02, p = 0.04). This represents roughly a 6.8% decrease in investigation evoked power across this time period (Control: 15.89 ± 2.76% increase over baseline; Cx36KO: 8.01 ± 2.16% increase).

## Discussion

Our findings reveal that Cx36, the principal gap junction protein expressed in GABAergic neurons, plays a crucial role in modulating high-frequency neural oscillations and evoked cortical dynamics, while exerting minimal effects on gross sleep/wake architecture. This extended characterization of Cx36KO mice demonstrates that electrical synapses formed by Cx36 are essential for the synchronization and integrity of thalamocortical circuits that underlie state transitions and cognitive processes—key domains disrupted in neuropsychiatric disorders such as schizophrenia.

### Functional role of Cx36 in oscillatory dynamics

Consistent with prior studies, we observed a robust reduction in spontaneous gamma and beta band activity in Cx36KO mice, reinforcing the idea that electrical synapses critically support high-frequency cortical oscillations [20, 36]. While gamma oscillations can be generated through chemical synaptic interactions alone, Cx36-containing gap junctions are thought to tighten interneuron synchrony, thereby enhancing gamma power [53]. Our data further show that this coupling is particularly important during behaviorally relevant and pharmacologically induced states, such as during ketamine administration and auditory steady-state stimulation. The diminished 40 Hz ASSR and blunted ketamine-induced gamma response in Cx36KO mice suggest that Cx36 facilitates both endogenous and evoked synchrony—deficits which closely parallel electrophysiological endophenotypes observed in schizophrenia [44].

### Sleep spindles and state transitions

Although global sleep architecture was largely preserved in Cx36KO mice, more nuanced impairments emerged in features tied to thalamocortical oscillatory regulation. Specifically, we observed reduced amplitude and duration of sleep spindles, as well as an attenuated sigma power surge during NREM-REM transitions. While this sigma attenuation was only observed during transitions, we do not believe that the Cx36KO effects on spindles is specific to these state change periods. Given the sparse nature of spindles during overall NREM, it is difficult for spindles to impact sigma power during overall NREM, and such effects are only reveal when examining periods in which spindle density is enhanced. These findings implicate Cx36 in the fine-tuning of thalamic reticular nucleus synchrony, where it is highly expressed and supports rhythmic burst firing [23, 38]. The selective reduction in spindle amplitude and pre-REM sigma power is particularly relevant given emerging evidence that such microstructural elements of sleep are crucial for memory consolidation and are disrupted in psychiatric illness [40, 41, 54].

### Deficits in evoked responses and cognitive biomarkers

Beyond spontaneous activity, we demonstrate that loss of Cx36 disrupts key translational EEG biomarkers, namely the MMN and ASSR. Cx36KO mice displayed diminished ERP amplitudes (notably N1 and P2) and reduced evoked power in the MMN paradigm. These impairments resemble hallmark features of sensory processing deficits in schizophrenia, where early auditory ERP components and gamma synchrony are consistently diminished [45, 48]. Patients with schizophrenia show a reduction in MMN that is positively associated with impaired cognition and poor functional outcome [47]. Importantly, our results suggest that these deficits may not solely stem from neurotransmitter imbalances but could also reflect fundamental disorganization of network connectivity arising from disrupted electrical coupling.

### Social cognition and behaviorally evoked gamma

In the social habituation paradigm, Cx36KO mice failed to exhibit normal reductions in investigatory behavior across repeated exposures to a novel mouse, indicative of impaired social recognition or habituation. This behavioral phenotype was accompanied by attenuated low gamma band activity elicited during social investigation, reinforcing the role of Cx36 in supporting top-down modulation and stimulus-induced synchrony in prefrontal networks. Given the importance of gamma oscillations in working memory, attention, and social cognition, our findings extend the relevance of Cx36 beyond basic physiology to complex behaviors disrupted in psychiatric disorders [4].

### Pharmacological sensitivity of gamma oscillations to NMDA antagonism

Our ketamine findings further underscore the dependence of gamma band activity on Cx36-mediated electrical coupling. While acute administration of subanesthetic ketamine robustly increased broadband gamma power in both WT and Cx36KO mice, the magnitude of this response was significantly blunted in knockout animals. This suggests that electrical synapses are not only integral to spontaneous gamma generation but also to the amplification of gamma synchrony elicited by NMDA receptor antagonism. Given that ketamine-induced gamma hyperactivity is often interpreted as a biomarker of cortical disinhibition and has been used to model aspects of psychosis, the attenuated response in Cx36KO mice implies a failure to engage typical synchronizing mechanisms under conditions of pharmacological excitation [43, 55]. These findings align with the hypothesis that Cx36-containing gap junctions may contribute to the pathological enhancement of gamma oscillations observed in psychosis models and suggest that disruption of electrical coupling may alter susceptibility to glutamatergic dysfunction.

### Therapeutic and translational relevance

Despite the absence of a direct genetic link between Cx36 and psychiatric disorders, our data suggests that Cx36-containing electrical synapses may represent a novel therapeutic target for modulating dysfunctional thalamocortical activity. This notion is supported by analogous cases in neuropharmacology—such as the orexin system’s role in narcolepsy and serotonin’s therapeutic relevance in depression despite GWAS studies failing to identify the receptors of these systems in disease [56–58]. Furthermore, electrical coupling is known to be modulated by intracellular Mg²⁺ levels, which are altered in several neurodegenerative conditions, presenting a potential avenue for pharmacological manipulation [59–61].

### Limitations and future directions

This study utilized global Cx36 knockout mice, limiting our ability to determine regional or circuit-specific contributions of electrical synapses to network function. Additionally, developmental compensation in the constitutive KO may obscure more acute or cell-type-specific roles of Cx36. Future work employing inducible and regionally targeted genetic tools will be essential to dissecting these mechanisms with greater precision. Furthermore, investigation of Cx36 modulation in models of psychiatric disease could clarify whether these effects are causal or compensatory.

Additionally, we note that all findings reported in this study were exclusively derived from EEG recordings from the frontal cortex and effects of the loss of Cx36 expression may vary across distinct brain regions. We believe the prevailing literature supports the use of frontal cortex EEG for all of the measures reported in this study. Speaking specifically to our acute ketamine findings, a number of published studies support the idea that acute ketamine leads to elevated gamma band activity in the rodent frontal cortex. Other studies suggest that this effect extends widely across the rodent cortex and even extends to subcortical structures including the thalamus [43, 62, 63]. Thus, we believe our finding that the acute ketamine induced rise in gamma band activity is impaired in Cx36 KO mice would be highly likely to occur across other cortical and subcortical regions. Regarding our findings with auditory evoked biomarkers (ASSR & MMN), prior studies have shown that both measures can be assed from frontal cortex EEG in rodents as well as other cortical regions. Interestingly, Hwang et al., [64] employed a high-density EEG approach to examine regional differences in ASSR response across the entirety of the mouse cortex and found that 40 Hz ASSR was most pronounced in the frontal cortex [64]. Despite this, we acknowledge that the effects of Cx36 KO may be in some measures have region specificity.

## Conclusion

In summary, our results provide compelling evidence that Cx36-mediated electrical coupling is critical for the integrity of cortical oscillatory dynamics and associated behaviors. The observed impairments in gamma synchrony, sleep spindles, and cognitive task performance in Cx36KO mice mirror key electrophysiological endophenotypes of neuropsychiatric disorders. These findings underscore the importance of electrical synapses in brain function and suggest that Cx36 may represent a novel point of intervention in the treatment of disorders marked by network-level dysregulation.

## Data Availability

All datasets and MATLAB analysis scripts used in this study will be made available upon reasonable request to the corresponding author.
